# Gastric and intestinal proteases resistance of chicken acidic chitinase nominates chitin-containing organisms for alternative whole edible diets for poultry

**DOI:** 10.1038/s41598-017-07146-3

**Published:** 2017-07-27

**Authors:** Eri Tabata, Akinori Kashimura, Satoshi Wakita, Misa Ohno, Masayoshi Sakaguchi, Yasusato Sugahara, Yoshihiro Kino, Vaclav Matoska, Peter O. Bauer, Fumitaka Oyama

**Affiliations:** 1 Department of Chemistry and Life Science, Kogakuin University, Hachioji, Tokyo, 192-0015 Japan; 2 Department of Bioinformatics and Molecular Neuropathology, Meiji Pharmaceutical University, Kiyose, Tokyo 204-8588 Japan; 3 Laboratory of Molecular Diagnostics, Department of Clinical Biochemistry, Hematology and Immunology, Homolka Hospital, Roentgenova 37/2, Prague, 150 00 Czech Republic; 4 Bioinova Ltd., Videnska 1083, Prague, 142 20 Czech Republic

## Abstract

Chitin, a polymer of *N*-acetyl-D-glucosamine (GlcNAc), functions as a major structural component in crustaceans, insects and fungi and is the second most abundant polysaccharide in the nature. Although these chitin-containing organisms have been suggested as novel animal feed resources, chitin has long been considered as indigestible fibers in the animal body. Recently, we reported that acidic chitinase (Chia) is a protease-resistant major glycosidase in mouse gastrointestinal tract (GIT) and that it digests chitin in the mouse stomach. However, the physiological role of Chia in other animals including poultry remains unknown. Here, we report that Chia can function as a digestive enzyme that breaks down chitin-containing organisms in chicken GIT. Chia mRNA is predominantly expressed in the glandular stomach tissue in normal chicken. We also show that chicken Chia has a robust chitinolytic activity at pH 2.0 and is highly resistant to proteolysis by pepsin and trypsin/chymotrypsin under conditions mimicking GIT. Chia degraded shells of mealworm larvae in the presence of digestive proteases and produced (GlcNAc)_2_. Thus, functional similarity of chicken Chia with the mouse enzyme suggests that chitin-containing organisms can be used for alternative poultry diets not only as whole edible resources but also as enhancers of their nutritional value.

## Introduction

The world’s consumption of meat is increasing with growing population. This process requires a continuous increase in animal feed production in the limited source of land and water^1^. A promising solution to these problems could be the introduction of proper living creatures that are rich in protein and have the ability for waste processing^2, 3^. About 1,900 species of insects are occasionally consumed by more than 2 billion people in some countries instead of meat protein^3^.

Chitin, a linear polymer of β-1, 4-linked *N*-acetyl-D-glucosamine (GlcNAc), is the second most abundant polysaccharide in nature and functions as a major structural component in crustaceans, insects, nematodes and fungi^4, 5^. Insects and fungi are ubiquitous organisms that have recently become attractive as potential novel animal feed resources because they are rich in protein^6^, high feed conversion efficiency^2^, and lower production of greenhouse gas and NH_3_ emissions than livestock^7^. As animal feed, these organisms are more acceptable for many consumers rather than ingesting them directly. Despite these advantages, there is a skepticism for using chitin-containing organisms as livestock diets^8^. Because chitin has long been considered as indigestible fibers in animal diets^9^, effects of chitin and chitin-containing organisms for the animal health and development are largely unknown^3, 8, 10^.

Chitinases hydrolyze the β-1, 4 glycoside bonds of chitin and belong to the family 18 of glycoside hydrolases^5, 11, 12^. Although mammals do not produce chitin, humans and mice express two active chitinases. Chitotriosidase (Chit1) was the first identified and purified mammalian chitinase^13–15^. Its levels are markedly increased in the plasma of patients with Gaucher disease, an autosomal recessive lysosomal storage disorder^13^. Acidic chitinase (Chia) was the second discovered mammalian chitinase^16, 17^. Chia has attracted substantial attention due to its expression fluctuations under certain pathological conditions. Significant changes in Chia mRNA and protein levels have been detected in asthma, allergic inflammation, ocular allergy, dry eye syndrome and gastric cancer^18–25^. Furthermore, genetic variants of Chia are associated with asthma in humans^25–28^.

Chia is highly expressed in normal mouse stomach tissues^16, 17, 29^. A robust peak of activity was observed at pH 2.0, suggesting that Chia can function as a digestive enzyme that breaks down chitin also as part of the host defense against chitin-containing pathogens in the gastric contents^16, 17, 29–32^. Recently we showed that Chia can function as a protease-resistant major glycosidase under gastrointestinal conditions in mouse^33^. However, the physiological role of the Chia in other animals including livestock remains unknown.

Chicken (*Gallus gallus domesticus*) is one of the major livestock animal species that produces eggs and meat as protein sources in the world. Here, we report that Chia can function as a protease-resistant digestive enzyme that breaks down chitin-containing organisms in normal chicken stomach, suggesting that such organisms can be included in poultry diets to enhance their nutritional value.

## Results

### Chia mRNA is highly expressed in glandular stomach in chicken

Chicken has two stomach tissues, glandular and gizzard. We investigated the Chia mRNA levels in the stomach tissues as well as other nine major chicken organs. We constructed a chicken standard DNA by ligating single cDNA of each: Chia, glyceraldehyde-3-phosphate dehydrogenase (GAPDH), pepsinogen A and H^+^/K^+^-ATPase (Supplementary Fig. S1) and performed Chia gene expression analysis using a quantitative reverse transcriptase-coupled PCR (qPCR) system as reported previously^29, 30, 33^.

**Figure 1:**
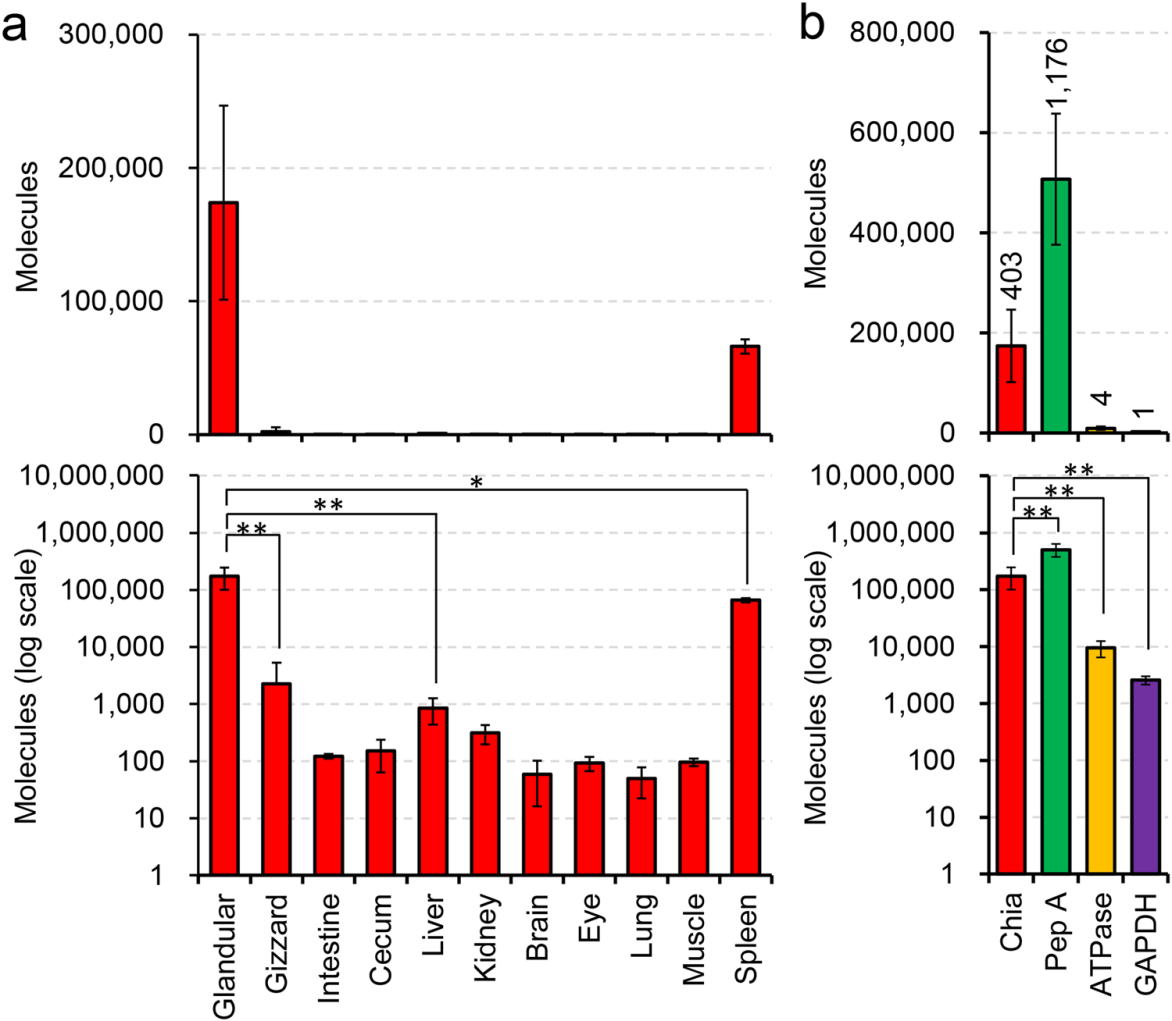
Chia mRNA is highly expressed in chicken glandular stomach tissue. Expression levels of Chia mRNAs in chicken tissues were quantified on the same scale by qPCR using the standard DNA (Supplementary Fig. S1). (**a**) Chia mRNA levels in chicken major eleven tissues. (**b**) The mRNA levels of four genes were quantified by qPCR in the glandular stomach. Y axis represents molecules per 10 ng of total RNA. The numbers in the figure indicate relative expression levels with GAPDH set at 1.0. Pep A, pepsinogen A; ATPase, H^+^/K^+^-ATPase. Values in (**a**,**b**) represent mean ± SD conducted in triplicate. *p < *0*.*05*, **p < *0*.*01*. P-values were determined using Student’s t-test.

We found that Chia mRNA was predominantly expressed in glandular stomach, followed by spleen, gizzard, liver and kidney with gizzard having 50 times lower expression than glandular stomach (Fig. 1a).

We next compared the expression levels of Chia with pepsinogen A, H^+^/K^+^-ATPase, and GAPDH. Pepsinogen A, the inactive zymogen, is autocatalytically converted into pepsin A at acidic conditions of the gastric fluid^34^. H^+^/K^+^-ATPase produces H^+^ to activate pepsinogen^35^. GAPDH is a well-known housekeeping gene that is constitutively expressed at high levels in most tissues and cells^36^.

We found that, similarly to Chia in mouse stomach, Chia mRNA was the second most abundantly expressed transcript in the glandular stomach tissue exceeded only by pepsinogen A while being significantly higher than those of GAPDH and H^+^/K^+^-ATPase (Fig. 1b). The relative expression levels of Chia, pepsinogen A and H^+^/K^+^-ATPase were 403, 1,176 and 4, respectively, when related to GAPDH (Fig. 1b). These results indicate that Chia mRNA is a major transcript in the chicken glandular stomach suggesting important physiological roles of the enzyme in the gastrointestinal tract (GIT) of chicken.

### Purification and characterization of chicken Chia from glandular stomach tissue

**Figure 2:**
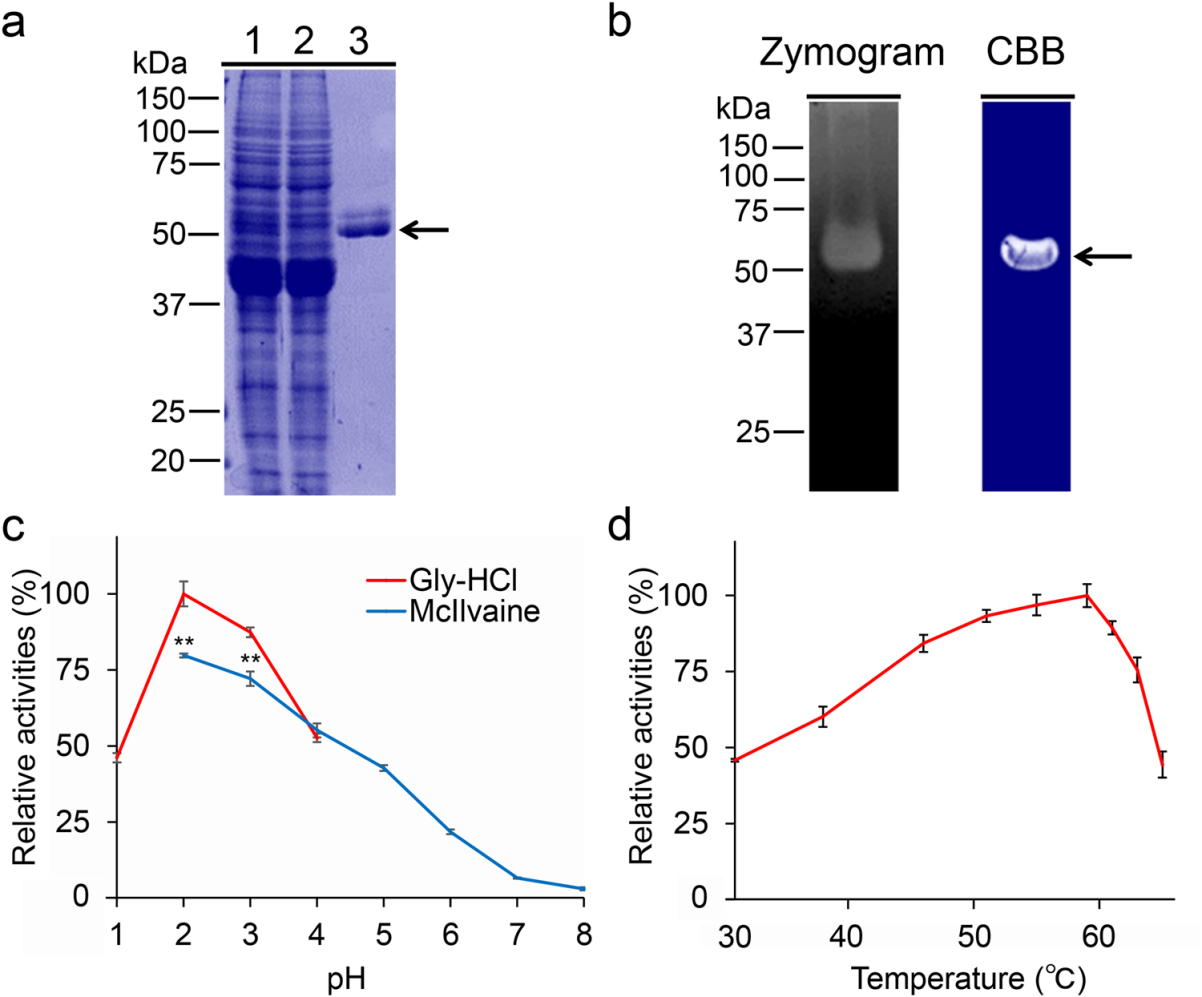
Purification and characterization of Chia from chicken glandular stomach. (**a**) Chia was purified from chicken glandular stomach tissues using chitin beads chromatography as described in Methods and analyzed by SDS-PAGE and visualized by Coomassie Brilliant Blue (CBB) staining. 1, extract; 2, flow- through; 3, purified enzyme. Purified Chia (1 μg of protein) was electrophoresed and visualized in the gel by CBB. (**b**) Zymogram of chicken Chia. After electrophoresis, the gel was analyzed by Zymography (left), followed by CBB staining (right). Zymography was performed as described in Methods. (**c**) Optimal pH and (**d**) optimal temperature for Chia activity. Values in (**c**,**d**) represent mean ± SD conducted in triplicate. **p < *0*.*01*. P-values were determined using Student’s t-test.

Chicken Chia is composed of an N-terminal catalytic domain (CatD) and a C-terminal chitin-binding domain (CBD)^37^. We purified Chia from chicken glandular stomach tissue using chitin beads chromatography through the chitin-binding activity of the CBD as described in Methods and summarized in Table 1. By SDS-PAGE and Coomassie Brilliant Blue (CBB) staining, we detected the enzyme as one major and one minor bands at 54 and 57 kDa, respectively (Fig. 2a). To further investigate these bands, we analyzed the N-terminal sequences as described in the Methods and found that in both bands, the amino acids corresponded to those in the mature form of chicken Chia (GenBank accession NP_989760.1): 54 kDa, YVLSXYFT; 57 kDa, YVLS. The mobility difference can be explained by posttranslational modifications of the protein such as phosphorylation and glycosylation.

**Table 1:** Purification of Chia from chicken glandular stomach.

Purification step	Total activity (mU)	Total Protein (mg)	Specific activity (mU/mg)	Yield (%)
Total soluble fraction	5845.7 ± 239.0	36.4 ± 4.6	160.6 ± 6.6	100
Chitin beads Flow through	660.1 ± 24.3	25.6 ± 1.2	25.8 ± 1.0	11.3 ± 10.2
Purified enzyme	440.8 ± 11.8	0.15 ± 0.04	2862.1 ± 326.4	7.5 ± 4.9

The purified protein was prepared from the 1 g of the glandular stomach as described in Methods.

Next, we analyzed whether the Chia possesses chitinolytic activity against ethylene glycol chitin at pH 2.0 in the gel. To maintain the chitinolytic activity, we used the sample buffer without SDS and reducing agent and loaded the sample onto the gel without heat denaturation as described in the Methods. Zymography analysis indicated a strong chitinolytic activity at around 54 kDa (Fig. 2b, left). Subsequent staining of the gel by CBB detected the 54 kDa band (Fig. 2b, right, arrow).

To gain more detailed insight, we examined the chitinolytic activity of the purified enzyme using 4-nitrophenyl *N*,*N*′-diacetyl-β-D-chitobioside (4-NP-chitobioside) as a substrate at 37 °C and pH 1.0–8.0 for 30 min. The pH optima were determined by monitoring the enzyme activity at indicated pH values in 0.1 M Gly-HCl (pH 1.0–4.0) and McIlvaine’s (pH 2.0–8.0) buffers. The highest activity was detected at pH 2.0 and it was decreasing in less acidic environments (pH 4.0–8.0) (Fig. 2c). Chitinolytic activity at pH 2.0 was higher when using Gly-HCl buffer as compared to McIlvaine’s buffer.

The effect of temperature on enzyme activity was determined in 0.1 M Gly-HCl buffer at pH 2.0 at temperatures ranging from 30 to 64 °C using same substrate for 30 min. As shown in Fig. 2d, the rate of the Chia-catalyzed reaction increased with rising temperature and reached a maximum level at 58 °C, then abruptly declined.

### Chia is stable in the presence of gastric and intestinal proteases

**Figure 3:**
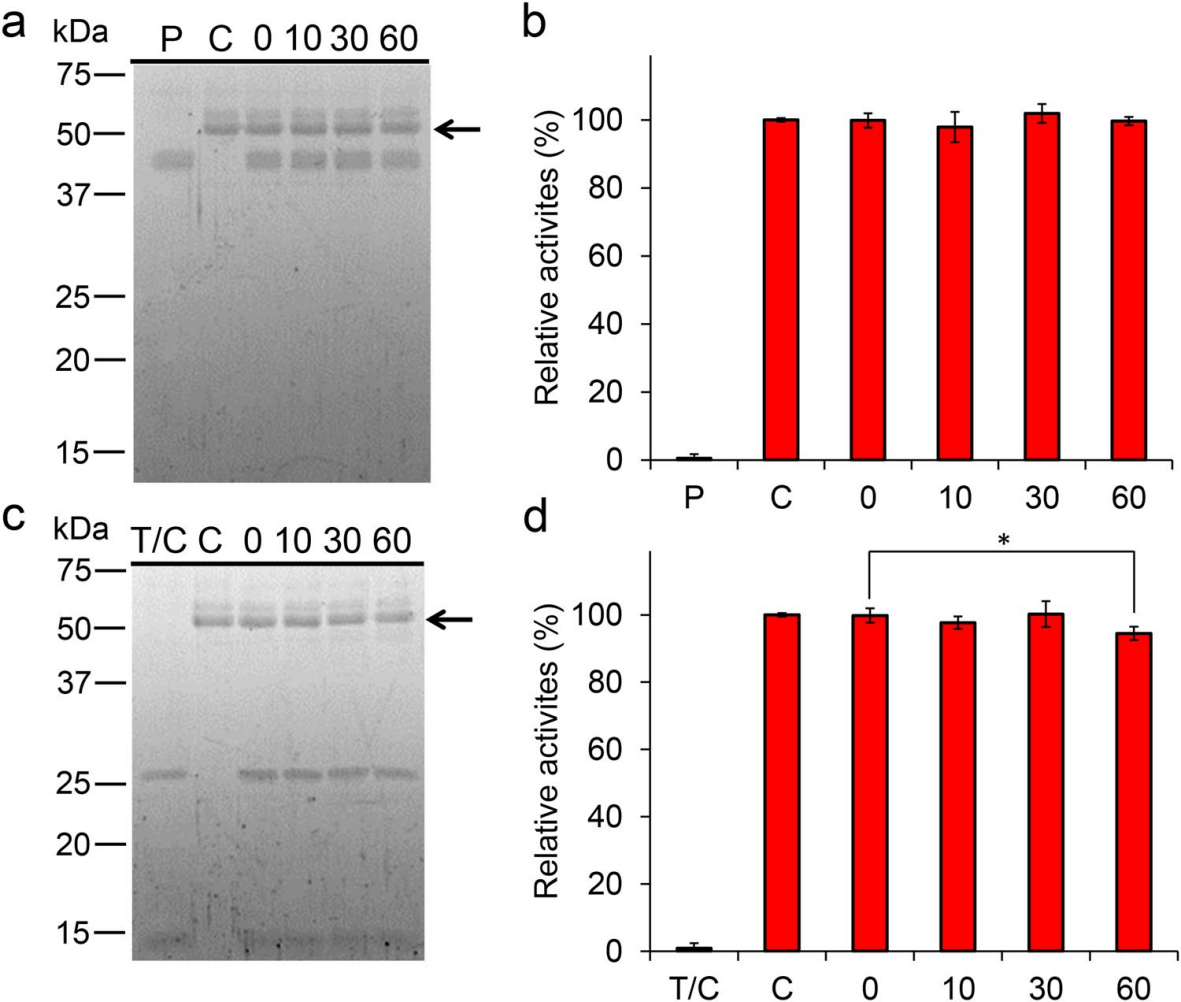
Functional stability of chicken Chia against gastrointestinal proteases. Purified Chia was incubated at 37 °C for 0, 10, 30, and 60 min under stomach-like (**a**,**b**) or intestine-like (**c**,**d**) environment in the presence of pepsin or trypsin and chymotrypsin at equal mass concentration of Chia. The samples were analyzed by SDS-PAGE and following (**a**,**c**) SYPRO Ruby staining, (**b**,**d**) chitinolytic activities. C, purified enzyme only; P, pepsin only; T/C, trypsin and chymotrypsin only; numbers, incubation time. Values in (**b**,**d**) represent mean ± SD conducted in triplicate. *p < *0*.*05*. P-values were determined using Student’s t-test.

To examine the protease resistance of chicken Chia, we first incubated the purified protein with pepsin at 1:1 ratio (1.5 µg/µL for each) under artificial stomach condition as described in Methods. As shown in Fig. 3a, we found that the levels of purified Chia were not changed within the 1 hour pepsin digestion at pH 2.0. Moreover, the chitinolytic activity of Chia was also not affected (Fig. 3b). When chitinolytic activity was set to 100% at time 0, nearly all the Chia activity was preserved after 1 hour incubation.

Next, we incubated purified Chia with trypsin and chymotrypsin at 1:1 ratio (1.5 µg/µL for each) under artificial intestinal condition. As shown in Fig. 3c, Chia was still present in the sample after 1 hour incubation. Similarly to the stomach condition, the chitinolytic activity was maintained during this incubation (Fig. 3d). These results revealed that Chia is resistant to gastric and intestinal proteases.

### Chia degrades chitin substrates in gastrointestinal conditions

**Figure 4:**
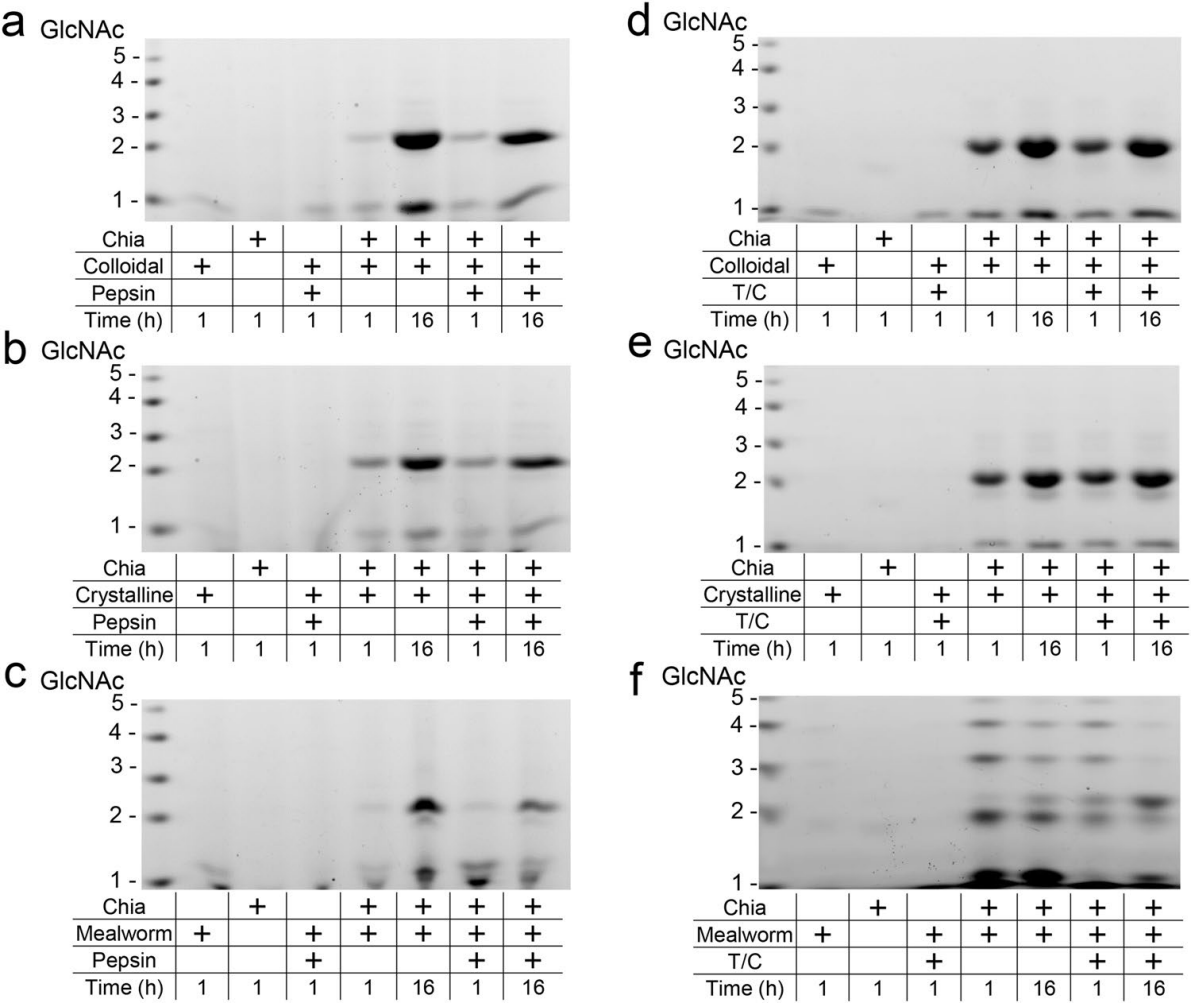
Chicken Chia degrades chitin substrates into (GlcNAc)_2_ in gastrointestinal condition. (**a**,**d**) Colloidal and (**b**,**e**) crystalline chitin substrates were degraded by purified Chia at (**a**,**b**) pH 2.0 or (**d**,**e**) pH 7.6 for 1 or 16 hours in the presence of pepsin or trypsin and chymotrypsin at 1:1 ratio. Shells of mealworm were also incubated with the enzyme for 1 or 16 hours under (**c**) stomach or (**f**) intestine condition. Resulting mono- and oligosaccharides were covalently labeled at their reducing end-groups with a fluorophore, followed by fluorophore-assisted carbohydrate electrophoresis (FACE). *N*-acetyl-chitooligosaccharides are shown in the left margin as standards.

We next tested whether chicken Chia can degrade chitin substrates in both gastric and intestinal environments. We incubated colloidal and crystalline chitin substrates with the purified enzyme under appropriate conditions. The resulting mono- and oligosaccharides were analyzed by fluorophore-assisted carbohydrate electrophoresis (FACE) as originally described by Jackson^38^ and recently improved by our group^39^. Purified Chia degraded colloidal and crystalline chitin substrates at pH 2.0 as early as after 1 hour incubation and produced primarily (GlcNAc)_2_ and a few GlcNAc fragments regardless on the presence of pepsin (Fig. 4a and b). Similarly, the enzyme degraded both substrates under intestine-like condition (pre-incubation at pH 2.0) (Fig. 4d and e). These results indicate that chicken Chia can degrade polymeric chitin in stomach as well as in the intestine.

We also assessed whether Chia can digest chitin-containing organisms in the presence of gastric proteases. Mealworm (*Tenebrio molitor*) larvae is one of the well-known edible insects as a source of protein for food purposes worldwide^3, 10, 40–44^. We incubated shells of mealworm with Chia with or without proteases under artificial gastrointestinal conditions for 1 or 16 hours. Chia degraded mealworm chitin and produced (GlcNAc)_2_ fragments in the presence of proteases (Fig. 4c and f). The product pattern after the degradation of shells of mealworm larvae differed from those using colloidal and crystalline chitin as natural substrates by the presence of longer oligosaccharides (Fig. 4f).

### Chia is stable and functional in chicken stomach extract

**Figure 5:**
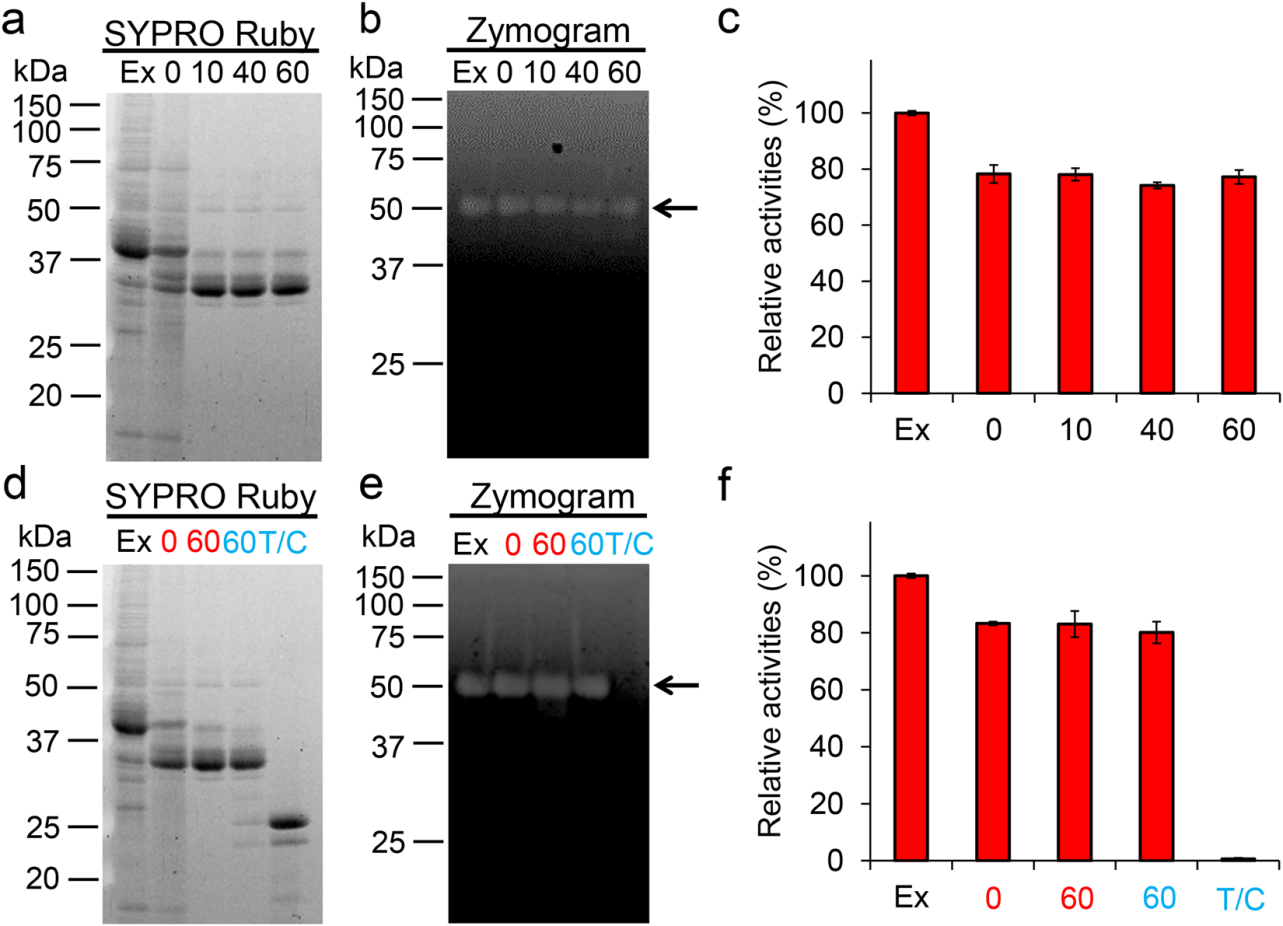
Functional stability of chicken Chia against endogenous pepsin. Soluble proteins obtained from chicken glandular stomach were incubated at pH 2.0 and 37 °C for 0, 10, 40 and 60 min (**a**–**c**). After the incubation, the samples were further incubated at pH 7.6 for 1 hour with trypsin and chymotrypsin in extract to the reaction (**d**–**f**). The samples were analyzed as follows: (**a**,**d**) SDS-PAGE and following SYPRO Ruby staining, (**b**,**e**) zymography, (**c**,**f**) chitinolytic activities. Ex, extract only; T/C, trypsin and chymotrypsin only; numbers, incubation time at pH 2.0 (red) or at pH 7.6 (light blue). Values in (**c**,**f**) represent mean ± SD conducted in triplicate.

Finally, we tested whether Chia functions as a chitin digestive enzyme in glandular stomach extract in the presence of endogenous pepsin and other proteins. We prepared soluble fraction from the stomach tissues and created artificial stomach and intestine condition followed by series of incubations at pH 2.0 and 37 °C for 1 hour, and subsequently under intestine-like neutral condition (pH 7.6) in the presence of 1:1 mixture of trypsin and chymotrypsin (at final concentration of 0.5 µg/µL) at 37 °C for 1 hour. We observed a marked decrease of total soluble protein after as early as 10 min of incubation at pH 2.0 (Supplementary Fig. S2), although several bands including that around 54 kDa bands remained unmodified within 1 hour of incubation (Fig. 5a). Also, subsequent incubation at pH 7.6 in the presence of trypsin and chymotrypsin resulted in further degradation of the total protein (Fig. 5d). Zymographic analysis indicated that Chia maintained its chitinolytic activity (Fig. 5b and e). Although the positions of the molecular weight markers in SDS-PAGE (Fig. 5a and d) and zymogram (Fig. 5b and e) were not matched at around 37 and 25 kDa, the corresponding bands on zymogram gels were not significantly affected during 1 hour incubation at pH 2.0 or pH 7.6. These results indicate that the chitinolytic activity of endogenous Chia remained virtually unchanged in gastrointestinal conditions.

**Figure 6:**
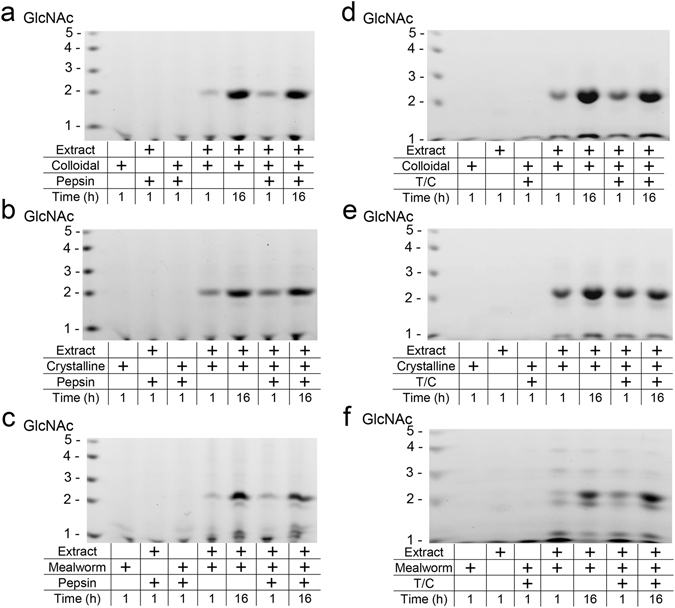
Chitin substrates and chitin-containing organisms are degraded by stomach extract. Degradation products were generated during incubation of (**a**,**d**) colloidal or (**b**,**e**) crystalline chitin and (**c**,**f**) mealworm sells with the extract proteins from stomach tissues. Incubation at pH 2.0 for 1 or 16 hours in the presence or being inhibited (adding pepstatin A) of pepsin (**a**–**c**) or at pH 7.6 for 1 or 16 hours in the presence of trypsin and chymotrypsin (pre-incubation at pH 2.0) (**d**–**f**). Degradation products were analyzed by the FACE method. Chitin oligomers are shown in the left margin as standards.

Next, we incubated chitin substrates with stomach extract at pH 2.0 for 1 hour followed by incubation at pH 7.6 for 1 or 16 hours in the presence of trypsin and chymotrypsin. The degradation products were analyzed by FACE method (Fig. 4). Chia in the stomach extract degraded chitin substrates and generated (GlcNAc)_2_ after 1 hour (Fig. 6a and b) as well as in the subsequent (pre-incubation at pH 2.0) incubation at pH 7.6 (Fig. 6d and e). We also incubated mealworm shells in the stomach extract and confirmed that chitin present in mealworm shells was degraded to (GlcNAc)_2_ by endogenous Chia after 1 or 16 hours incubation in both stomach and intestine environments (Fig. 6c and f).

## Discussion

In this report, we showed that Chia mRNA was predominantly expressed in normal chicken glandular stomach. We purified this enzyme from the stomach using chitin affinity chromatography. Activity of purified Chia has optimum at pH 2.0 and 58 °C. It is resistant to pepsin and trypsin/chymotrypsin digestion. Furthermore, we showed that shells of mealworm, a representative chitin-containing organism, are degraded by Chia in the presence of digestive proteases. These results may introduce chitin-containing organisms as whole edible diets for poultry^2, 8, 10^.

Mammals have Chia and Chit1 genes in their genomes while birds, including poultry (chicken, duck, turkey and geese), do not have Chit1 genes according to the NCBI genome database. This is a major distinction between mammals and birds^45^. Thus, we focused only on Chia analysis in this report.

We quantified the Chia mRNA transcripts in eleven major tissues of normal chicken on the same scale using qPCR as described previously^29, 30, 33^. Chia mRNA level was remarkably high in glandular stomach, followed by spleen, gizzard, liver and kidney. These findings are essentially consistent with a previous study using *in situ* hybridization which showed Chia mRNA expression in oxyntico-peptic cells in chicken glandular stomach^37^. Also, it has been reported that broiler chickens can secrete chitinase in the gizzard^46^. Our results show that Chia mRNA was the second most abundantly expressed transcript in the glandular stomach tissue being exceeded only by pepsinogen A and its level was significantly higher than those of housekeeping and other gastric genes.

We have reported that Chia is predominantly expressed in normal mouse stomach, where Chia mRNA level is comparable to that of pepsinogen C^29, 33^. There were differences in Chia mRNA levels between mouse inbred strains of BALB/c and C57BL/6J mice, where C57BL/6J mice express about 10-fold more Chia mRNA than BALB/c mice^29^. It has been known that there are differentially expressed genes whose expression levels can be altered around 10-fold in different genetic backgrounds^47^. The expression levels of Chia mRNA in normal chicken glandular stomach in this study were comparable to that in BALB/c^29^, but 10 times lower than that in C57BL/6 J mice^29, 33^. Thus, there are similarities in the Chia mRNA levels between chicken and BALB/c mice stomach tissues, indicating that Chia mRNA is constitutively expressed in normal mouse and chicken stomach tissues.

The digestive system in chicken is different from that of mammals. Chicken transfers food via esophagus to the glandular stomach, where it is biochemically digested by pepsin at pH 1.5–3.5. In gizzard, the food is then physically decomposed into smaller particles by muscle contractions^48, 49^. Purified Chia has optimal activity at pH 2.0 and is stable against gastric proteases. Thus, this novel knowledge on Chia indicates that it plays important roles in biochemical digestion of chitin-containing diets in the GIT.

Chicken Chia degraded shells of mealworm larvae in the presence of digestive proteases and produced (GlcNAc)_2_. The product patterns were slightly differed from those resulting from colloidal and crystalline chitin degradation. We detected longer oligosaccharides as well as multiple bands around the (GlcNAc)_2_ after mealworm larvae processing. During our recent investigation, we found that specific fragments with slower-than-expected mobility as defined by chitin oligosaccharide markers were generated at pH 5.0~8.0 as by-products of in the fluoresceinated reaction for FACE analysis^39^. We established an improved method for chitin oligosaccharides detection that suppresses the by-products generation in the fluoresceinated reaction^39^. Because we applied our improved method in this study, we cannot fully explain the occurrences of the multiple bands.

However, following possibilities can be considered: *Serratia marcescens* chitinases can degrade partially deacetylated chitin resulting in chitooligosaccharides with unexpected molecular weight^50, 51^. Such observations have been described regarding human Chit1^52^. Indeed, we confirmed formation of longer chitooligosaccharides from chitosan (71% degree of deacetylation) by chicken Chia (Supplementary Fig. S3). There were the bands with similar mobilities in samples derived from crystalline chitin, mealworm larvae shell and chitosan, while others were not consistent with chitosan and GlcN oligomer markers (Supplementary Fig. S3). Thus, distinct chitooligosaccharides can be produced from partially deacetylated crystalline chitin or mealworm shell by chicken Chia.

Increased Chia expression levels at both mRNA and protein levels have been reported to be related to pathological conditions, such as asthma and allergic inflammation^18, 19^. One may think that chitin in the chitin-containing organism could increase Chia expression suggesting adverse effects on chicken health. However, transgenic mice overexpressing Chia showed no signs of spontaneous inflammation, indicating that Chia attenuates chitin-induced allergic innate immune responses by enzymatically degrading chitin in lungs^19, 53^. Using Chia-deficient mice, it has also been shown that Chia is a constitutively produced enzyme necessary for chitin degradation in the airways to maintain lung function^54^ and that Chia plays a critical role in the development of type 2 immunity to the gastrointestinal nematodes in the host GIT^55^. As for stomach tissues in humans, it has been known that Chia levels are not different or decreased in the inflammation of stomach cancer caused by *Helicobacter pyroli* ^23, 24^. This suggests that Chia itself is not harmful, but necessary for mice and humans’ health and that inflammation does not cause Chia overexpression in stomach tissues. Thus, constitutive high expression of Chia seems to be safe also in chicken.

Moreover, chitin-containing organisms have been practically used for poultry diets as alternative antibiotics or maze/soybean based feed^56^, because chitin-containing organisms including fly larvae (maggot), mealworm and cricket are rich in protein, fats, minerals and vitamins^10, 40, 57^. Dietary supplementation with dried mealworm increased daily gain and daily feed intake and IgG and IgA levels in chicken, while reducing feed conversion ratio and mortality^44^. Other study showed that mealworm-based meal can be used to replace soybean meal in broiler diets during the growing period without negative effects on diet palatability^43^.

Our present results showed that Chia degraded chitin in mealworm shells while producing (GlcNAc)_2_ even in the presence of pepsin or trypsin and chymotrypsin. These results indicate that chitin fragments may serve as antibiotics or have a potential as probiotics and could result in improvement of the overall health and performance of chicken^44^. In addition, Chia is constitutively and predominantly expressed in chicken glandular stomach tissues. Indeed, chitin-containing organisms including insects are natural diet for birds. Based on our and others’ observations, we do believe that chitin-containing organisms can be used as alternative novel diets for chicken. However, further investigation on the effects of chitin degradation products on the health, development and immunity of poultry are needed to practical use of chitin-containing organisms as protein, mineral, carbon and nitrogen source for chicken.

## Methods

### Chicken stomach tissues

We purchased normal chicken glandular and gizzard stomach tissues from Funakoshi Co., Ltd, which were dissected from white leghorn and quickly frozen on dry ice and kept at −80 °C.

### RNA and cDNA preparation

The Chicken Total RNA Panel (Zyagen) was used to examine the distribution of Chia transcript in normal chicken tissues. In addition, total RNA was isolated from glandular and gizzard stomach tissues of white leghorn chicken (Funakoshi) using TRIzol Reagent (Invitrogen) per manufacturer’s instructions and reverse transcribed into cDNA essentially as described previously^33^.

### Selection of primer pairs for qPCR

Primers for qPCR were designed using PrimerQuest Input (Integrated DNA Technologies) and evaluated their suitability based on whether they gave single products, as reflected by a single melting temperature (Tm). PCR reactions (final volume 13 µL) contained 2 × SYBR Green Master Mix (Brilliant II SYBR Green QPCR Master Mix, Agilent, Santa Clara, CA, USA), 2.7 ng of chicken cDNA or appropriate amount of the external standards (see below) and 2.5 pmol of primers for Chia, pepsinogen A, H^+^/K^+^-ATPase and GAPDH. The PCR reactions were performed using Mx3005 P QPCR System (Agilent) as follows: an initial denaturation and polymerase activation step for 10 min at 95 °C, followed by 40 cycles of denaturation at 95 °C for 30 sec, annealing at 55 °C for 30 sec and polymerization at 72 °C for 10 sec. Melting curves were generated after amplification. The primers’ sequences are listed in Supplementary Table 1.

### Construction of the DNA standard and qPCR

Construction of the four genes standard DNA containing the genes of major proteins in stomach was described previously^33^. Coding sequences of Chia (GenBank accession number NM_204429.1, nucleotides 414–492 of the Chia cDNA), GAPDH (GenBank accession number NM_204305.1, nucleotides 75–174), pepsinogen A (GenBank accession number NM_204878.1, nucleotides 324–436) and H^+^/K^+^-ATPase (GenBank accession number NM_204418.1, nucleotides 272–368) template DNA, which was consisted of the regions amplified by the selected forward and reverse primers as described above, were synthesized and inserted into pTAKN-2 vector by Eurofins Genomics. After ligation, these genes were amplified using the forward primer 5′-GCTGGACATTGACTGGGAATA3′ and the revers primer 5′-GTAATCTGGCTCGTAGGGATTG-3′. The standard DNA (389 bases; see Supplementary Fig. S1) was prepared by PCR reamplification from the plasmid DNA using the same primers and was thereafter used as the standard DNA for qPCR.

### Purification of the chicken Chia

Chicken glandular stomach tissue (1 g) was homogenized in 10 volumes of ice-cold TS buffer [20 mM Tris-HCl (pH7.6), 150 mM NaCl] containing protease inhibitor (Complete Mini, Roche) using a Teflon/glass homogenizer and centrifugation at 15,000 g for 10 min at 4 °C, and the supernatants were kept. A chitin beads (New England Biolabs) column was prepared by mixing 3 mL chitin beads, followed by equilibration with TS buffer. The extracts were applied onto the column. The chitin beads column was sealed and gently inverted at 4 °C for 1 hour. After extensive washing with TS buffer without protease inhibitors, bound chitinase was eluted from the column with 8 M urea, which was subsequently removed by desalting using PD-10 (GE healthcare) equilibrated with TS buffer without protease inhibitors. Protein concentrations were determined by the Bradford Protein Assay (Bio-Rad) using the BioPhotometer Plus UV/Vis photometer (Eppendorf), with bovine serum albumin as a standard. Chia unit definition was also reported previously^31^.

### SDS-polyacrylamide gel electrophoresis and CBB staining or SYPRO Ruby staining

The obtained protein fractions were performed using standard SDS-polyacrylamide gel electrophoresis (PAGE)^58^, followed by Coomassie Brilliant Blue R-250 (Sigma-Aldrich) or SYPRO Ruby staining (Thermo Fisher Scientific) and analyzed using the Luminescent Image Analyzer (ImageQuant LAS 4000, GE Healthcare). We used All Blue (Bio-Rad Laboratories) as molecular weight markers.

### N-terminal protein sequencing

The purified protein fraction was separated by 12.5% SDS-PAGE, electroblotted onto a polyvinylidene fluoride (PVDF) membrane (Immobilon-P, Millipore) and then stained with Coomassie brilliant blue (CBB). The 54 kDa and 57 kDa bands on the membrane were excised and subjected to N-terminal sequencing by automated Edman degradation using Procise 492 (Applied Biosystems).

### Zymography

Zymography was performed using standard SDS-PAGE gels^58^ except for containing 0.1% ethylene glycol chitin (Wako Pure Chemical Industries). We used the sample buffer without SDS and reducing agent in the sample buffer and loaded the samples onto the gel without heat denaturation. After electrophoresis, the gel was incubated in 50 mM Gly-HCl (pH 2.0) containing 1% (V/V) Triton-X-100 at 37 °C for 1 hour. After that, gels were submerged in 50 mM Tris-HCl (pH 7.6) containing 1% (V/V) Triton X-100 at 37 °C overnight. After refolding, the gel was stained with freshly prepared 0.01% (W/V) Calcofluor white M2R (Sigma-Aldrich) in 50 mM Tris-HCl (pH 7.6). After 30 min-gentle shaking, the brightener solution was alkalized for emission. The gels were analyzed using the Luminescent Image Analyzer. Then, the gel was stained with CBB.

### Chitinase enzymatic assays

Chitinolytic activity was determined using the synthetic chromogenic substrate, 4-nitrophenyl *N*,*N*′-diacetyl-β-D-chitobioside (Sigma-Aldrich) essentially as described previously^31^. All enzymatic reactions for the determination of optimum pH and temperature were conducted in a volume of 50 μL as described previously^31, 32^.

### Protease treatments

Purified enzyme (1.5 µg) was incubated with the same amount of pepsin A (Worthington) (1.5 µg) in 0.1 M Gly-HCl (pH 2.0) or 1:1 mixture of the trypsin (Sigma-Aldrich) and chymotrypsin (Sigma-Aldrich) (1.5 µg) based on mass concentration in 0.1 M Tris-HCl (pH 7.6) at 37 °C for 0, 10, 40 and 60 min. After incubation, protease inhibitors were added and acid conditions were neutralized by addition of 1 M Tris-HCl (pH 7.6).

### Glandular stomach extract preparation

Soluble fraction was prepared from chicken glandular stomach tissues as described above except that the protease inhibitors were omitted in the homogenizing buffer. The homogenates were centrifuged at 15,000 g for 10 min at 4 °C, and the supernatants were used as chicken proteins extract. Gly-HCl buffer (pH 2.0) was added at final concentration of 0.1 M. After the pre-incubation at 37 °C for 0, 10, 40 or 60 min, protease inhibitors were added. After incubation under the conditions of pH 2.0 at 37 °C, the solutions were neutralized by addition of 1 M Tris-HCl (pH 7.6). Then, 1:1 mixture of the trypsin and chymotrypsin (10 μg) was added to the reaction mixture. The reaction mixtures were further incubated at 37 °C for 1 hour at pH 7.6.

### Mealworms

Jumbo mealworm (*Tenebrio molitor*) larvae were purchased from local commercial supplier in Japan. The worms were sacrificed and immersed in deionized water and blow-dried for measuring the dry weight. We used the shells containing connective tissue as chitin-protein polymer substrates.

### Degradation of colloidal and crystalline chitin substrates and mealworm larvae shells by purified Chia

Colloidal chitin was prepared from shrimp shell chitin (Sigma-Aldrich), as described previously^31, 33, 59^, and used as a substrate to determine the chitinase activity. The shell of mealworm larvae is also used as a chitin-protein polymer substrate. The enzymatic reactions under stomach or intestine condition using colloidal chitin (at a final concentration of 1 mg/mL), crystalline chitin (1 mg) and mealworm larvae shell (1 mg) were performed in a volume of 50 µL containing 4 µL of 400 mU/mL purified enzyme with equal amount of pepsin A at pH 2.0 and 37 °C for 1 hour or following incubated with trypsin and chymotrypsin at pH 7.6 and 37 °C for further 1 hour. Chitin fragments generated in the gastrointestinal conditions were analyzed by fluorophore-assisted carbohydrate electrophoresis (FACE) as originally described by Jackson^38^ and recently improved by our group^39^. *N*-acetyl chitooligoaccharides (Seikagaku Corporation) were used as a standard.

### Degradation of colloidal and crystalline chitin substrates and mealworm larvae shell by soluble protein

The enzymatic reactions in gastrointestinal conditions using colloidal chitin, crystalline chitin and mealworm shell as substrates were performed in a volume of 50 µL containing 4 µL of 400 mU/mL soluble protein from chicken glandular stomach and reacted as described above. To repress the protease activity in soluble protein, we added pepstatin A (Sigma-Aldrich) to final concentration of 1 µg/µL^60^. Generated chitin fragments were analyzed described above.

### Statistical analysis

Biochemical data were compared by Student’s t-test.

## Electronic supplementary material

Supplementary Information
