# Supplementary material for: Gastric and intestinal proteases resistance of chicken acidic chitinase nominates chitin-containing organisms for alternative whole edible diets for poultry
Source: Sci Rep. 2017 Jul 27;7:6662. doi: 10.1038/s41598-017-07146-3 (PMC5532213; doi:10.1038/s41598-017-07146-3)
Supplement: Supplementary file 1 — Supplementary Information [file 41598_2017_7146_MOESM1_ESM.pdf]

Supplementary Information

Gastric and intestinal proteases resistance of chicken acidic chitinase nominates chitin-containing organisms for alternative whole edible diets for poultry

Eri Tabata[1], Akinori Kashimura[1], Satoshi Wakita[1], Misa Ohno[1], Masayoshi Sakaguchi[1], Yasusato Sugahara[1], Yoshihiro Kino[2], Vaclav Matoska[3], Peter O. Bauer[3, 4], Fumitaka Oyama[1]

[1]Department of Chemistry and Life Science, Kogakuin University, Hachioji, Tokyo 192-0015, Japan, [2]Department of Bioinformatics and Molecular Neuropathology, Meiji Pharmaceutical University, Kiyose, Tokyo 204-8588, Japan, [3]Laboratory of Molecular Diagnostics, Department of Clinical Biochemistry, Hematology and Immunology, Homolka Hospital, Roentgenova 37/2, Prague 150 00, Czech Republic, [4]Bioinova Ltd., Videnska 1083, Prague 142 20, Czech Republic

a.

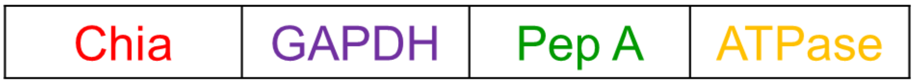

b.

GCTGGACATTGACTGGGAATACCCTGGATCAAAGGGCAGCCCTT
CTCAGGACAAAGGT**CTCTTCACCGTCCTTGTTCAGCAACGGATT**
TGGCCGTATTGGCCGCCTGGTCACCAGGGCTGCCGTCCTCTCTG
GCAAAGTCCAAGTGGTGGCCATCAA**TGATCCCTTCATCGATCTG**
AACTGTGGGTGCCCTCTATCTATTGCAAAAGCTCGGCCTGCAGC
AACCACAAACGCTTTGACCCCTCCAAGTCCTCAACCTACGTGAG
CACCAAC**GAAACCGTCTACATCGCCTACCCTGTACTACCTGGCC**
TTCTACGTGGTGATGACTGGGATCTTCGCCCTCTCCATATACTC
CCTAATGAGGACGGT**CAATCCCTACGAGCCAGATTAC**

Figure S1. The single standard DNA used for qPCR. (a). Schematic representation of the standard DNA used for qPCR. (b). Nucleotide sequence of the single standard DNA. The single standard DNA, 389 nucleotides long, contained cDNA fragments of Chia, GAPDH, pepsinogen A and H^+/K^+-ATPase in a one-to-one ratio. The PCR-target regions are shown in underline. Red, Chia; purple, GAPDH; green, pepsinogen A; orange, H^+/K^+-ATPase.

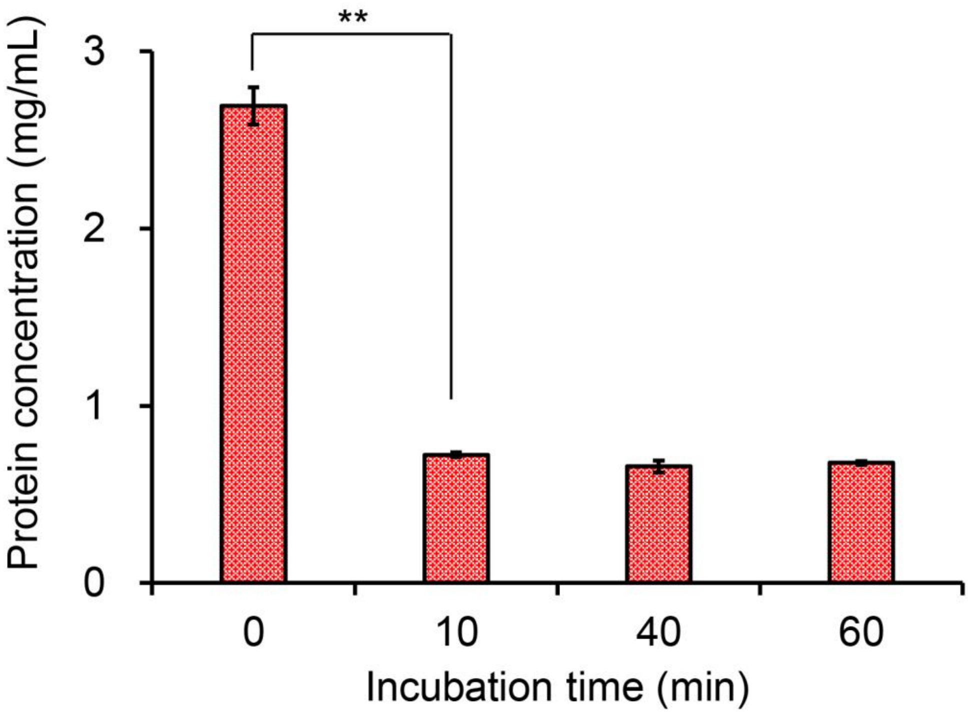

Figure S2. A marked decrease of total soluble protein by incubation at created artificial stomach condition. The soluble protein fractions were incubated at pH 2.0 and 37°C. Total protein levels were quantified by Bradford method. We observed a marked decrease of total soluble protein after as early as 10 min of incubation at pH 2.0. Values represent mean ± SD conducted in triplicate. **$p < 0.01$. P-values were determined using Student's t-test.

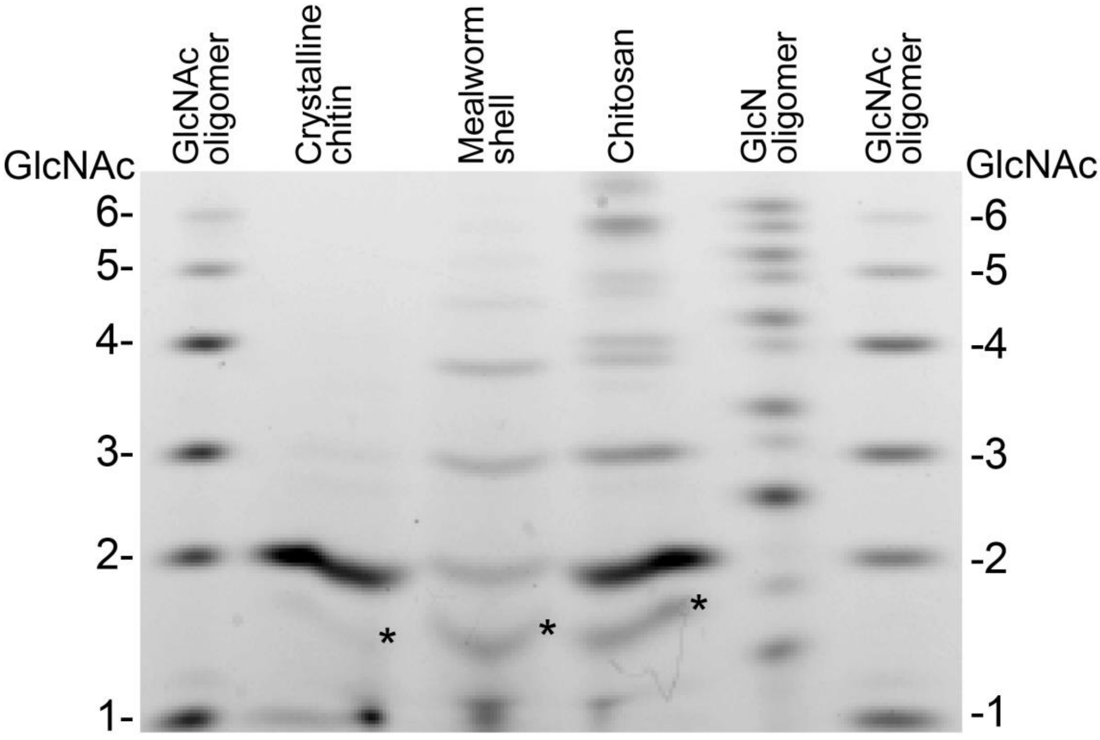

Figure S3. The multiple bands around the $(GlcNAc)_2$ and longer chitooligosaccharides can be derived from chitosan. Chitosan (71% degree of deacetylation, Funakoshi Co., Ltd) as well as crystalline chitin and mealworm larvae shells were incubated with chicken Chia and analyzed as described in Fig. 4e and f. Glucosamine (GlcN, Tokyo Chemical Industry Co., Ltd) and GlcN oligomers [$(GlcN)_{2\text{-}6}$, Seikagaku Corporation] were used as GlcN oligomer markers. The GlcNAc oligomers are shown in the both margins as standards. Asterisks show the bands with similar mobilities in samples derived from crystalline chitin, mealworm larvae shell and chitosan digested by chicken Chia.

Table S1. List of qPCR primers.

Gene	Forward primer	Reverse primer
Chia	GCTGGACATTGACTGGGAATA	CTGAACAAGGACGGTGAAGAG
GAPDH	CAACGGATTTGGCCGTATTG	GTTCAGATCGATGAAGGGATCA
Pepsinogen A	TGTGGGTGCCCTCTATCTAT	GTAGGCGATGTAGACGGTTTC
H^+/K^+-ATPase	CCTGTACTACCTGGCCTTCTA	GTAATCTGGCTCGTAGGGATTG
